# Higher levels of total pepsin and bile acids in the saliva as a possible risk factor for early laryngeal cancer

**DOI:** 10.2478/raon-2014-0020

**Published:** 2015-03-03

**Authors:** Maja Sereg-Bahar, Ales Jerin, Irena Hocevar-Boltezar

**Affiliations:** 1 University Medical Center Ljubljana, Department of Otorhinolaryngology and Head & Neck Surgery, Ljubljana, Slovenia; 2 University Medical Center Ljubljana, Institute for Clinical Chemistry and Biochemistry, Ljubljana, Slovenia

**Keywords:** laryngopharyngeal reflux, gastric acid, pepsin, bile acids, laryngeal carcinoma

## Abstract

**Background:**

Gastroesophageal reflux is suspected to be an etiological factor in laryngeal and pharyngeal cancer. The aim of this study was to establish, using a non-invasive method, whether laryngopharyngeal reflux (LPR) appears more often in patients with early laryngeal cancer than in a control group.

**Patients and methods:**

We compared the pH, the level of bile acids, the total pepsin and the pepsin enzymatic activity in saliva in a group of 30 patients with T1 laryngeal carcinoma and a group of 34 healthy volunteers.

**Results:**

The groups differed significantly in terms of levels of total pepsin and bile acids in the saliva sample. Higher levels of total pepsin and bile acids were detected in the group of cancer patients. No significant impact of other known factors influencing laryngeal mucosa (*e.g*. smoking, alcohol consumption, and the presence of irritating substances in the workplace) on the results of saliva analysis was found.

**Conclusions:**

A higher level of typical components of LPR in the saliva of patients with early laryngeal cancer than in the controls suggests the possibility that LPR, especially biliary reflux, has a role in the development of laryngeal carcinoma.

## Introduction

Gastroesophageal reflux (GER) disease is caused by the pathological retrograde flow of gastric contents into the esophagus. In laryngopharyngeal reflux (LPR), the gastric contents pass the upper esophageal sphincter and reach the pharynx and the larynx. It is believed that LPR is never physiological, because the mucosa of the upper respiratory tract is more sensitive to gastric contents than the esophageal mucosa.^1^

In LPR, the mucosa of the upper aerodigestive tract is exposed to the effects of gastric contents (acid and non–acid reflux). In the case of even minimal biliary reflux, the non-acid reflux consists of pepsin and conjugated bile acids. Acid and non-acid reflux have a synergistic effect on the mucosa of the upper aerodigestive tract.^2^

Laryngeal cancer is mentioned as one of the possible extraesophageal complications of GER disease by some authors.^1^ In addition, an association between LPR and laryngeal carcinoma has been suggested, though the causality remains unproven. LPR prevalence among patients with laryngeal cancer has been found to be as high as 67%.^3^–^8^

In the present study we tried to identify which components of LPR are present in the saliva of patients with early laryngeal cancer. For this purpose, the levels of the various components of LPR (pepsin, gastric acid and bile acids) in saliva were determined in patients with early laryngeal cancer and in a control group of healthy volunteers.

## Patients and methods

### Patients and controls

**FIGURE 1.**
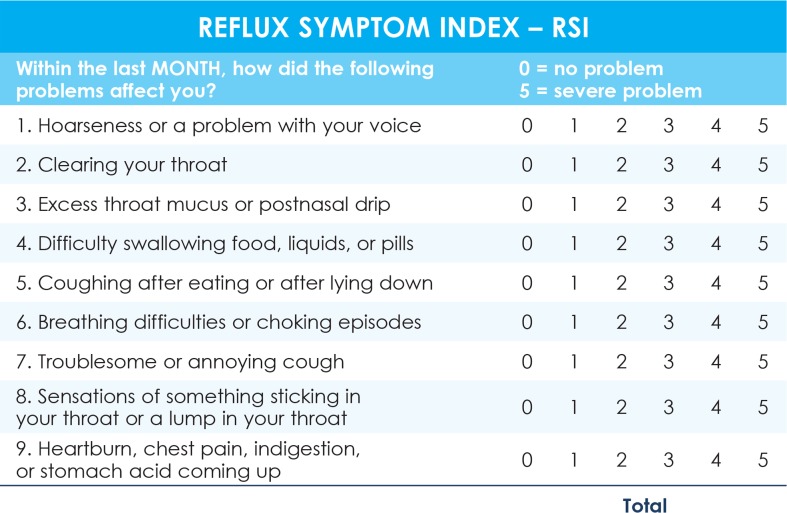
Reflux Symptom Index questionnaire.

Thirty successive patients with early laryngeal cancer (T1 squamocellular carcinoma of the vocal folds) admitted to a tertiary center for their first treatment between 2011–2012 and 34 successive healthy volunteers matched by age +/− 5 years (accompanying persons of patients at the general otorhinolaryngological outpatient clinic having no major health problems according to the results of a questionnaire about past pulmonary, cardiac, gastroenterological, neurological, and renal diseases, i.e. the control group) were included in the study. Only those subjects who were willing to undertake the proposed examinations were included in the study. The study protocol was approved by the National Medical Ethics Committee/number 44/04/04. In both groups, the data on LPR symptoms were obtained through a standardized Reflux Symptom Index (RSI) questionnaire regarding any extraesophageal reflux problems that had been present for at least 5 years (Figure 1).^9^ An RSI score > 13 is considered to be a result of LPR.^9^ The participants were asked to fill in a questionnaire about typical GER symptoms (heartburn, regurgitation) and other factors causing similar symptoms as LPR, *i.e*. smoking habits (smoker/nonsmoker; for smokers: number of cigarettes per day, years of smoking), regular alcohol consumption (intake of more than 140 mg of alcohol per week in men or 70 mg of alcohol per week in women, years of regular alcohol intake), and exposure to irritating substances (wood dust, concrete dust, asbestos, acid fumes, gas vapors) at the workplace (exposed/not exposed). The data about the localization of the T1 laryngeal tumor (the anterior half of the vocal fold, the posterior half of the vocal fold or the entire vocal fold) were obtained from the medical documentation of the patients.

### Biochemical analyses

Saliva samples were taken from all the participants, one from each participant. Participants spit their saliva (2ml) through a straw into a testing tube at least two hours after their last meal. Samples were frozen and kept at a temperature below −20 °C until analysed. The pH, the level of total pepsin, the pepsin enzymatic activity and the level of bile acids of the samples were measured.

The pH measurement was conducted using pH indicator papers (Siemens Healthcare, Erlangen, Germany) and the results were rounded up to the nearest half of a unit. The concentrations of bile acids in the saliva samples were measured via the enzyme method using an Olympus AU600 biochemical analyser (Beckman Coulter, Brea, CA, USA) and appropriate reagents (Alere Ltd, Stockport, UK). Bile acids were converted into 3-keto-steroids and thio-NADH in the presence of thio-NAD and 3-α-hydroxy steroid dehydrogenase enzymes (3-α HSD). The speed of the reaction of origin of thio-NADH at 405 nm, which is proportional to the bile acid concentration in the sample, was measured. Bile acid concentration in saliva is given in μmol/l.

An ELISA (Enzyme-Linked Immunosorbent Assay) specific for human pepsin (USCN Life, Wuhan, China) was used to determine the total pepsin concentration in the samples. The sample was immobilized on a solid support. A specific antibody linked to a special enzyme was applied over its surface so it could bind to the antigen (pepsin). A substance containing the enzyme’s substrate was added. The subsequent reaction produced a detectable signal, a color change in the substrate. Pepsin concentration in saliva was measured in μg/l.

The protease enzyme activity was established with a colorimetric test of enzyme activity (PDQ Protease Assay, Protease Determine Quick Test, Athena Sciences, Baltimore, USA). The test substrate contained proteins bound to a fluorescein pigment. In the presence of active pepsin the substrate changed colour. The protease activity was determined spectrophotometrically or fluorometrically. The increase in optical density of the sample was proportional to the increase in pepsin enzymatic activity. Enzyme activity was measured in numbers of kilo units per liter - kU/L.

### Statistics

The general data (gender, age), the results of the questionnaire about factors influencing the larynx (smoking habits, alcohol consumption, and the presence of irritating substances in the workplace), the total score of the RSI questionnaire, and the results of the saliva samples’ analysis were compared for the group of T1 laryngeal cancer patients and the control group. In order to assess the influence of other factors on the pH value, the levels of bile acids, total pepsin and pepsin enzymatic activity, correlations between the results of the biochemical analysis of the saliva samples and the known etiologic factors for laryngeal cancer, gender and age were performed in the T1 laryngeal cancer group and in the control group. The SPSS 19.0 statistical package (SPSS Inc., Chicago, IL, USA) was used to perform the analyses. The data were analyzed using the χ^2^-test, the Fisher exact test, Pearson’s correlation, the Spearman rank correlation, the t-test, and the nonparametric Mann-Whitney test. All the statistical tests were two-sided and a p-value of £ 0.05 was considered to be statistically significant.

## Results

Compared to the laryngeal carcinoma patients, there were more females (p = 0.055) and significantly fewer smokers and those exposed to irritating substances in the control group. No difference in the mean age was observed between the two groups (Table 1). Among patients, the cancer appeared in the anterior half of a vocal fold in 30 patients (53.6%), in the posterior half of a vocal fold in 3 patients (5.4%), and over the entire vocal fold in 23 patients (41%).

In the group of T1 laryngeal patients, 8 subjects (26.7%) had problems with heartburn and 6 subjects (20%) with regurgitation. There were 8 subjects (26.7%) with at least one typical GER symptom in the T1 laryngeal group. In the control group only one person (2.9%) had problems with regurgitation. In addition, there were 7 patients (23.3%) with an RSI score above 13 whereas none of the controls had an RSI score above 13 (Table 1).

**FIGURE 2.**
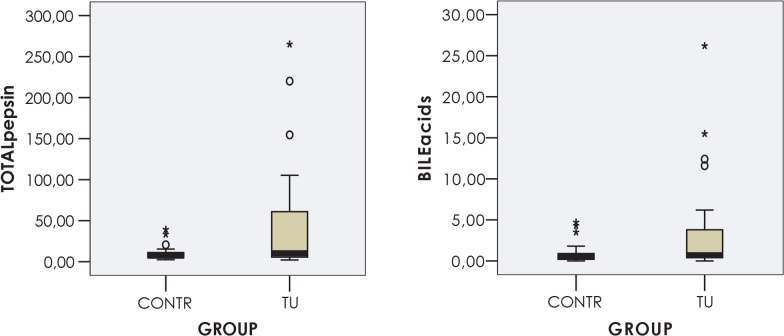
Distribution of total pepsin and bile acid concentration in the saliva samples of patients with T1 laryngeal cancer. TU = laryngeal cancer (N = 30); CONTR = control group (N = 34).

A significant difference between the groups was detected with regard to the level of total pepsin and bile acids in saliva. The mean levels of total pepsin and bile acids in the saliva were more than four times higher in patients with early laryngeal cancer than in the subjects of the control group. No difference in pH or pepsin enzymatic activity was recorded between the two groups (Table 2, Figure 2).

Using correlation analysis we tried to establish whether gender, age, smoking, alcohol intake and exposure to irritants have an impact on the biochemically determined levels of pH, total pepsin, bile acids, and pepsin enzymatic activity in the laryngeal cancer group and in the control group. No significant correlations were found in either group (Table 3).

## Discussion

The results of our study established a much higher mean level of bile acids and total pepsin in the saliva of the patients with T1 squamous cell laryngeal carcinoma than in the control group of healthy volunteers. The scores of the standardized RSI questionnaire were also significantly higher in the group of T1 laryngeal carcinoma patients than in the controls. As pepsin and bile acids are excreted only in the gastrointestinal tract below the level of the pharynx, all these results indicate greater retrograde movement of the gastric contents to the level of the upper aerodigestive tract in patients with early laryngeal carcinoma than in the control group.

Our results confirm the results of other studies that detected LPR in a considerable number of patients with laryngeal cancer using 24-hour ambulatory double pH monitoring.^3^–^8^ However, the exact mode of influence of LPR on laryngeal mucosa has yet to be revealed. The first of the studied parameters, the pH value, was very similar in the group of patients with T1 laryngeal cancer and in the controls. We believe that a much larger volume of saliva excreted in the oral cavity in comparison with the volume of LPR was the reason for such result.

Pepsin enzymatic activity did not differ significantly in the two groups, which could be attributed to the rather similar pH values found among patients and controls. Pepsinogen, the enzymatically inactive precursor of pepsin, is secreted by gastric mucosa cells and transformed into enzymatically active pepsin by gastric acid. However, at a mean pH value of 7.0–7.2, which was the level measured in our patients and controls, pepsin is mostly inactivated.^10^

In our study, the level of total pepsin was significantly higher in the T1 laryngeal cancer patients than in the controls. Total pepsin includes active and inactivated pepsin and is, as previously mentioned, a good indicator of LPR. An immunologic pepsin assay of combined sputum and saliva was determined to be 100% sensitive and 89% specific for detection of extraoesophageal reflux (based on pH-metry).^11^

The level of bile acids was also significantly higher in our patients with T1 laryngeal cancer than in the control group. Several studies have shown a significant correlation between subtotal gastrectomy and an increased risk of the development of laryngeal carcinoma. Such patients have pyloric dysfunction, which enables large quantities of biliary reflux to reach the upper aerodigestive tract, causing the occurrence of malignant disease. It has been estimated that 20 years after gastric surgery, patients have a ten times higher risk of laryngeal cancer than the population who had not undergone gastric surgery.^6^, ^12^

Smoking, excessive alcohol consumption and occupational exposure to irritating substances are all known etiological factors for laryngeal cancer^13^–^15^, which case relative high incidence of the disease.^16^ It is also known that alcohol intake increases the distal oesophagus exposure to acid reflux.^17^ High body mass index (BMI) and longer duration of re-flux symptoms are also risk factors for the occurrence of LPR.^19^ BMI data were not obtained from our participants, therefore we cannot discuss the role of BMI in the aetiology of LPR in our study.

In the present study, the group of patients with T1 laryngeal cancer and the control group differed with regard to smoking and exposure to irritants in the workplace, as expected. Because correlation analysis excluded any relationship between these risk factors and the results of biochemical analyses in our participants, it is suggested that the differences in levels of total pepsin and bile acids in saliva between cancer patients and controls are the result of LPR rather than the activity of the abovementioned factors.

Only subjects considered healthy according to data from the questionnaire about their possible diseases were included in the control group. Of these, only one control subject showed a typical GER symptom (*e.g*. regurgitation). The participants were asked about any extraoesophageal symptoms (RSI protocol) lasting for at least five years. None of the controls had an RSI score above 13. Nevertheless, a certain level of total pepsin, bile acids and pepsin enzymatic activity was also found in the saliva of the majority of the control group. It is possible that the presence of pepsin and bile acids is a sign of flow of the gastric contents to the oesophagus and up to the level of the oral cavity even in completely asymptomatic patients, but the concentrations are lower. A study including a large asymptomatic group would help to determine what levels of the pepsin and bile acids in saliva constitute a normal range.

Laryngeal cancer typically develops in the membranous portion of the vocal folds and rarely in the posterior part of the larynx. It has been suggested that malignant alteration is rare in the posterior part of the larynx where carbonic anhydrase is expressed in the mucosal cells and neutralizes any acid, thereby protecting the mucosa.^3^ Actually, it has not yet been proven that the gastric contents come in contact with the mucosa of the anterior part of the vocal folds. It has been shown, however, that the hypersensibility of the laryngeal mucosa to the chemical (acid) stimulation of reflux causes the adduction of the vocal folds and closure of the larynx^20^, thus not allowing the reflux to reach the anterior parts of the vocal folds. There are no data on the effect of biliary reflux on laryngeal sensibility. In the present study, malignant tumors appeared on the posterior part of the vocal fold in only 3 patients. In all other patients, cancers extended over the anterior half or over entire vocal fold. This raises the question of how reflux can directly act on vocal folds’ mucosa causing malignant changes.

The main drawback of our study is that there were some differences between the cancer patient group and the control group. A different gender ratio in both groups was noticed. There were 83% men and 17% women in the T1 cancer group, and 62% men and 38% women in the control group. Although the difference was noticeable, it was not statistically significant.

The groups also differed with regard to smoking habits and exposure to irritating substances in the work place. Smoking is a known risk factor for laryngeal cancer. Therefore it was expected that the majority of the laryngeal cancer patients would be smokers. The data on smoking, alcohol intake and workplace were obtained because these factors can cause similar laryngeal and pharyngeal symptoms as LPR. When the correlations between smoking, irritating substances in the workplace, and the results of saliva testing were performed, no significant results were found.

A larger number of included subjects would give firmer proof of the role of LPR in the etiopathogenesis of laryngeal cancer. Our results for the group of 30 patients and 34 controls suggest a possible role for LPR. In order to really prove that LPR is a risk factor for laryngeal cancer, only those patients with laryngeal cancer who are nonsmokers and not exposed to noxious substances in the workplace should be included in the study.

Furthermore, it remains unclear how pepsin and bile acids, which can cause malignant alteration in the mucosa, come in contact with the vocal folds.

## Conclusions

Significantly higher levels of LPR components in the saliva of patients with T1 laryngeal carcinoma may indicate that LPR plays a role in the development of laryngeal carcinoma.

A further study of LPR’s role in the etiology of laryngeal carcinoma is necessary, especially in patients with no other known cancer risk factors, *i.e*. smoking, alcohol consumption or exposure to irritating substances in the workplace.

## Figures and Tables

**TABLE 1.** Historical data and the results of the RSI questionnaire for patients with early laryngeal carcinoma and the control group

**Parameter**	**T1** **N = 30**	**C** **N = 34**	**p**
**Gender – male / female**	25 / 5	21 / 13	0.055
**Age** (mean /standard deviation – years)	58.8 / 8.1	57.5 / 15.1	0.676
**RSI score** (mean /standard deviation)	10.8 / 4.3	3.9 / 3.1	**0.000**
**Smokers**	29	16	**0.000**
**Number of cigarettes/ day** (mean /standard deviation)	24 / 10.4	16.5 / 7.7	0.005
**Smoking years** (mean /standard deviation)	34.6 / 9.6	22.6 / 11.8	0.000
**Regular alcohol consumers**	10	17	0.178
**Duration of alcohol consumption** (mean /standard deviation – years)	29.5 / 12.9	29 / 12.2	0.914
**Occupational exposure to irritating substances**	28	6	**0.000**

T1 = patients with T1 laryngeal carcinoma; C = control group; N = number of subjects

**TABLE 2.** Results of the saliva analysis for patients with T1 laryngeal carcinoma and the control group

**Parameter**	**T1** **N = 30**	**C** **N = 34**	**p**
**Saliva pH**	5.0 – 8.5	5.0–8.5	0.382
/ range mean / standard deviation	7.0 / 0.8	7.2 / 0.8	
**Bile acids**	0–26.2	0–4.7	**0.031**
- μmol/L / range mean /standard deviation	3.4 / 5.9	0.9 / 1.2	
**Total pepsin**	2.1–265	2.4–39.28	**0.044**
- μg/L / range mean / standard deviation	44.6 / 66.8	9.6 / 8.1	
**Pepsin enzymatic activity**	0.1–35.3	0.4–30.5	0.204
- kU/L / range mean /standard deviation	7.7 / 6.9	6.2 / 6.3	

T1 = patients with T1 laryngeal carcinoma, C = control group, N = number of participants

**TABLE 3.** Correlations between gender, age, smoking, alcohol intake, presence of irritating substances in the workplace, and the pH value, the levels of bile acids, total pepsin and pepsin enzymatic activity in the patients with T1 laryngeal carcinoma (N = 30)

**Parameter**	**Saliva pH**	**Bile acids**	**Total pepsin**	**Pepsin enzymatic activity**
**Gender**		U=54.5	U=73	U=36
	p=0.753	p=0.776	p=0.146	p=0.1,000
**Age**	r=0.096	rho=−0.014	rho= 0.033	rho=0.080
	p=0.619	p=0.943	p=0.864	p=0.693
**Smoking**		U=14.5	U=13	U=3
	p=1,000	p=0.952	p=0.905	p=0.199
**Alcohol intake**		U=93.5	U=85	U=56
	p=0.820	p=0.945	p=0.646	p=0.145
**Irritating substances in the workplace**		U=94	U=78	U=64
	p=0.102	p=0. 963	p=0.435	p=0.382

U = Mann Whitney U; r= Pearson’s coefficient; rho = Spearman’s coefficient

**TABLE 4.** Correlations between gender, age, smoking, alcohol intake, presence of irritating substances in the workplace, and the pH value, the levels of bile acids, total pepsin and pepsin enzymatic activity in the control group (N = 34)

**Parameter**	**Saliva pH**	**Bile acids**	**Total pepsin**	**Pepsin enzymatic activity**
**Gender**		U=65	U=136.5	U=117
	p=0.873	p=0.054	p=0.811	p=0.313
**Age**	r=0.348	rho=−0.072	rho= −0.245	rho=0.027
	p=0.053	p=0.699	p=0.170	p=0.891
**Smoking**		U=99	U=122.5	U=82
	p=0.099	p=0.425	p=0.651	p=0.315
**Alcohol intake**		U=126	U=144.5	U=130
	p=0.364	p=0.812	p=0.759	p=0.275
**Irritating substances in the workplace**		U=54.5	U=37	U=43
	p=0.053	p=0.571	p=0.097	p=0.326

U = Mann Whitney U; r = Pearson’s coefficient; rho = Spearman’s coefficient
